# Health and Well-Being in Late Life: Gender Differences Worldwide

**DOI:** 10.3389/fmed.2019.00218

**Published:** 2019-10-10

**Authors:** Sara Carmel

**Affiliations:** Department of Public Health, Faculty of Health Sciences, Center for Multidisciplinary Research in Aging, Ben-Gurion University of the Negev, Beer Sheva, Israel

**Keywords:** gender differences, health, well-being, old age, international differences

## Abstract

Maintaining health and quality of life and decreasing the number of years lived with disabilities in old age are among the main challenges of aging societies worldwide. This paper aims to present current worldwide health-related gender inequalities throughout life, and especially in late life, as well as gender gaps in social and personal resources which affect health, functioning and well-being. This paper also addresses the question of whether gender gaps at younger ages tend to narrow in late life, due to the many biological and social changes that occur in old age. Based on international data regarding these gender gaps and the trends of change in personal resources and health-related lifestyles in the more and less developed nations, conclusions regarding future changes in gender gaps are presented, along with practical implications for future improvements in women's health and well-being.

## Introduction

One of civilization's main achievements in the last century is the unprecedented prolongation of life. However, societal achievements often breed new needs and challenges. Although years of healthy life expectancy have increased, the rise in the absolute size and share of people aged 60 and older, and especially of those aged 80 and older, is accompanied by increasing numbers of people living with chronic diseases and disabilities for longer years [e.g., (1)], leading to a significant burden on families and on society as a whole. As a result of these developments, maintaining good health and quality of life and decreasing the number of years lived with disabilities in old age are challenges of aging societies worldwide (1). The need to address these challenges draws from humanitarian values embedded in the cultures of democratic countries, as clearly presented in the US Declaration of Independence: “We hold these truths to be self-evident, that all men are created equal, that they are endowed by their Creator with certain unalienable Rights, that among these are Life, Liberty and the pursuit of Happiness” (US Declaration of Independence, 1776). Another pressing need to address this challenge derives from the foreseen threat to the economies of countries facing rapid growth in a large, mostly unproductive sector of the population, and increasing expenses for people requiring medical and social services [e.g., (2)]. Hence, understanding why certain aging population groups are more susceptible than others to infirmity, poor quality of life, and dependence on families and society is of the utmost importance. In this context, it is well-established worldwide that although women comprise more than half of the world's population and generally live longer than men, they also live more years of their lives with functional limitations (1, 3–6). Furthermore, when compared to men, older women also score significantly lower on most indicators of subjective well-being and mental health (7–12).

Gender differences are part of human existence, expressed both in biological structure and functions. However, it appears that beyond the biological differences it is culture with its social structure including division of gender-related roles, societal functions and social status that has been a more influential factor in determining gender differences in quality of life. In the Old Testament we read God's words to woman: “In pain you shall bring forth children,” (Genesis 3:16), while to men—“By the sweat of thy brow you shall eat bread” (Genesis 3:19). For thousands of years, a strong paternalistic approach has dominated human beliefs and behavior worldwide, including division of social roles, social power and status. Preservation of the paternalistic dominance of men has been served by cultural beliefs and the societal regulations deriving from them. In most countries, societal barriers have been established to prevent women from attaining positions of social power within family and society. Thus, in many societies, women have been barred from acquisition of personal resources, such as education and economic means which contribute to the development of social status, economic independence and societal influence. Although some of these barriers have been lifted in recent decades (mainly in Western countries), current cohorts of elderly people have lived most of their lives in paternalistic societies [e.g., (13)].

Gender-related societal role division, with its special demands and expectations from both genders, has been one of the domains that have hampered gender equality. However, old age is a stage of life characterized by significant transitions for both genders, including changes in societal roles and gender-related expectations and status within family. Men are not expected to continue working, and in many societies are obliged to retire from work. Women no longer have to bear and raise children. Furthermore, in many countries governments provide equal old age pensions to men and women, thus equalizing women's status with that of men, to a certain degree. In addition to the changes in income and diminishing societal and familial obligations in old age, both genders undergo a series of biological processes due to increased susceptibility and exposure to disease, hormonal changes, and decline in physical and mental functioning.

In this paper, we will address the following question: Considering all of the aging-related changes in both genders and the prolonged aging process, does the gap in well-being between men and women narrow in old age? Based on international data sets and the literature, we aim to support one of two opposing hypotheses regarding gender differences in health and well-being in old age. One is the divergence hypothesis—indicating persistence of women's disadvantages, and the other is the convergence hypothesis—indicative of changes toward increasing gender equality.

This paper is based on an interdisciplinary literature review of internationally recognized statistical data, and articles (including meta-analyses) on gender differences in health, published since the 1970's in biomedical and social science journals and books. We shall begin with an overview of gender differences along the life course, focusing on two basic aspects of quality of life—health and subjective well-being.

## Health and Well-Being

According to the World Health Organization's declaration signed in 1946 by 61 nations, “Health is a state of complete physical, mental and social well-being and not merely the absence of disease or infirmity” [(14), p. 100]. In other words, health is perceived as a multifaceted phenomenon, which in addition to lack of physical and mental disease, also includes dimensions of quality of life as perceived and facilitated by society. Therefore, health is determined by people's genetic composition, as well as by physical, cultural and societal environmental conditions. Since the second half of the last century, unparalleled developments in medicine, medical technology, sanitation, nutrition and standard of living have significantly enhanced human health and life expectancy. Cultural knowledge and beliefs regarding determinants of illness and health, and derived changes in lifestyles have also played an important role in preventing disease and promoting health.

According to the World Health Organization's definition of health, a comprehensive evaluation of health must be based on a variety of measures. Physical health is usually evaluated by life expectancy, lack of disease and infirmity. Mental health is generally evaluated by lack of mental illness and dementia, as well as by psychological characteristics and coping resources, such as self-esteem, self-efficacy, hardiness, and sense-of-coherence. Social well-being is assessed by socioeconomic status, family status, social involvement and social support.

Quality of life is usually assessed by objective measures of these domains. However, people who are ranked similarly on objective measures of quality of life may differ in their subjective evaluations of it. Furthermore, repeated findings from various countries show that subjective evaluations of health and well-being are better predictors of elderly persons' survival than objective measures of health, including physicians' evaluations (15–17). These findings have resulted in extensive research focusing on subjective evaluations of health and well-being, especially among older adults [e.g., (18)].

Subjective well-being (SWB) is an expression of the way one views his/her life in general. It is evaluated by measures, such as subjective evaluation of health, satisfaction with life, morale, worthiness of life, happiness, and will-to-live, along with lack of depression, anxiety and loneliness. Studies indicate that health and SWB are intercorrelated. There are some indications that SWB affects physical health and physical functioning rather than the other way around (19–23). However, both are determined by the specific combination of people's biological and social structures, as well as by their lifestyles and inherited and/or acquired personal resources. Personal resources can be acquired during the life course, and can positively affect individuals' health and well-being in old age. Cultural and societal conditions can enhance or impede these processes for different individuals as well as for entire social groups. A comparison between genders regarding the ability to accumulate personal resources in various nations is a good way to evaluate such societal influences worldwide.

## Gender Differences in Personal Resources and Social Roles Related to Health and Well-Being

In modern and open societies, education and income are two of the most important personal resources for social mobility, and are also significant determinants of outcomes in health and well-being. For many centuries, women have had lower levels of education than men worldwide. Although there are indications that this gap has narrowed over the years, it continues to exist in numerous countries. For example, while literacy rates for women aged 15+ worldwide increased from 76.4% in the year 2000 to 82.6% in the year 2016, these ratios increased among men from 86.6% in the year 2000 to 89.8% in the year 2016 (24, 25).

Gender gaps in education level vary by country. According to United Nations (UN) classifications, countries are divided into three groups based on their socio-economic status including criteria, such as gross domestic product (GDP), per capita income, level of industrialization, standard of living, life expectancy, and literacy level. The more or “most developed” nations include mainly North American and Western European countries, as well as Australia, New Zealand, and Japan. The “less developed,” or “developing” nations include countries, such as Namibia, South Africa, China, Malaysia, India, Brazil, and Bolivia. The “least developed” countries include the poorest nations in Africa, Asia and the Middle East, such as Afghanistan, Sudan, Ethiopia, and Yemen (26). Levels of education of both genders are by definition significantly higher in the more developed nations than in the less developed nations. With time, in many countries, the gender-related education gaps either narrow or disappear completely in the younger age groups (ages 35–44). Nevertheless, currently in most OECD countries, older men have higher levels of formal education than older women (27).

Regarding income, the percentage of women participating in the labor force is typically lower than that of men worldwide (13, 28). This pattern of social order continues to be a significant barrier for women's independence and equality, but has gradually been changing since the second half of the last century. In recent decades growing percentages of women acquire higher education, join the labor force, and increase their presence in high societal positions, mainly in the more developed nations. Despite significant improvements for women in level of education and participation in the labor force, women still encounter a “glass ceiling”—a widely used metaphor referring to the invisible societal barriers to women's progress toward employment equity. This phenomenon occurs even in the most modern societies and at all levels of employment, from the largest corporations to the lowest-paying jobs at the bottom of the labor ladder (13, 29, 30). Among employed persons, women are less likely than men to hold positions of power, have job security, authority, autonomy, and opportunities for advancement (13, 27, 31, 32). As a result, women generally have lower incomes (33). This situation derives from many persisting cultural and societal barriers. Women are still disadvantaged in dominant orientations toward gender roles and in central beliefs regarding gender-related capabilities—a social phenomenon termed “sexism.” For instance, based on a longitudinal US study conducted from 1979 to 2005, Judge and Livingston (34) conclude: “Although gender role attitudes in terms of beliefs that people hold about the proper roles for men and women at home and at work are becoming more similar for the two genders, traditional role orientation continues to exacerbate the gender wage gap” (p. 994).

All of these facts indicate that when comparing economic status of men and women, one finds lower percentages of women participating in the labor force, and significantly higher ratios of women holding part-time and low status positions. Generally, women are also employed for fewer years over their life course and earn lower wages than men do. As a result, in old age, lower percentages of elderly women receive pensions from work places, and if women do receive them, their pensions are lower than those of men, and therefore, their socio-economic status is inferior to that of men (1, 35). The gender gaps in education and income continue to exist in old age and they affect older adults' health and well-being (36), as will be further discussed below.

### Associations Among Education, Income, and Health

Numerous studies demonstrate the associations among the personal resources of education income and health in old age. The causal relationship between low income and poor health has often been reported in medical sociology literature (37, 38). Among elderly Americans, level of education was found to be a good predictor of life expectancy and active life expectancy (39, 40). A significant association between socioeconomic status and women's mortality has also been reported in an Israeli study (41). Ross and Wu summarized these interrelationships: “High educational attainment improves health directly, and it improves health indirectly through work and economic conditions, social-psychological resources, and healthy lifestyle” ((42), p. 719). In line with Ross and Wu's findings and conclusion, an Israeli study conducted during a long physicians' strike showed that education is not only a means for gaining social power through higher positions and income. Education is also an important resource for handling any life crisis more effectively and efficiently including health problems, due to greater accessibility of informal pathways to information, knowledge, and networks of professionals and influential people in positions of power (43).

### Gender Differences in the Role of Informal Caregiving

In addition to women's disadvantages in education and income with the resulting negative influences on health and well-being, one of the most prominent factors affecting women's socioeconomic status, health, and well-being is women's social role of nurturing children and providing caregiving to ailing family members. For centuries, this traditional role has been one of the barriers to women's ability to extend their contribution from the family unit to society as a whole, thus negatively affecting their status in society. In recent decades, this traditional role has changed somewhat. On the one hand, in many societies there is a decrease in number of children, but on the other hand, the burden of caregiving has significantly increased due to the prolonged years of life with infirmity of old family members. Women usually occupy the role of family caregivers, often for long periods while taking care of a number of family members (children, grandchildren, parents, and spouses) at the same time and/or successively. Furthermore, in modern families women continue to perform these caregiving tasks across their life course along with multiple roles at home and the additional role of working outside the home.

In fact, women comprise the majority of informal caregivers in general and, particularly, for people suffering from dementia (1). A US comparison between men and women caregivers found that women comprise 70% of primary caregivers. Furthermore, women are more likely to invest more weekly hours in caregiving, and to perform a greater amount of the more difficult personal caregiving tasks than men. Women also continue their caregiving role into old age, a fact that exposes them more than men to the heavy multifaceted burden and stressors related to these tasks while being physically and emotionally worn out themselves (44). The role of nurturing and caregiving thus prevents women from fully participating in the work force while they are young and raising children; it negatively affects their social status and income, and exposes them to continuous physical and emotional burdens throughout the life course.

The growing numbers of frail older people in aging societies have increased public awareness of the hazards of the caregiving role. Studies have shown that primary caregivers of older people have poor quality of life (45, 46), and are a group at high risk for nutritional deficiencies (47), stress and depression (48–52), and general morbidity and mortality (52, 53), especially if they themselves are elderly persons.

It thus appears that the accumulated disadvantages in education and income, along with the traditional caregiving role, which have long been impediments to women's social status and mobility at young ages, continue to exist in old age, when their negative implications for health and well-being are significantly intensified. This can be seen in existing gender gaps in health which are often to the disadvantage of older women.

## Gender Differences in Health

The health of populations can be evaluated by a number of measures, including life expectancy and mortality, life expectancy with disability, prevalence of diseases and of risk factors, and level of physical and mental functioning.

### Life Expectancy

Life expectancy—the average number of years from birth a person can expect to live, assuming the current age-specific mortality rates in the population continue to exist—is one of the most common measures used for comparatively assessing the health of a populations. Differences among nations in life expectancy are an outcome of a diversity of factors. In addition to genetic composition, environmental, cultural, and societal conditions, such as housing, sanitation, standard of living, and medical and preventive services influence people's quality of life, morbidity and life expectancy.

In general, women outlive men around the globe. However, both life expectancy and the gender discrepancy in life expectancy vary across countries according to their socioeconomic ranking (54). Life expectancy is highest in developed nations, declines in the less developed nations, and is lowest in the least developed countries. Similarly, the gender gap in life expectancy is greatest in developed nations and smaller in less developed and least developed nations (55, 56). Worldwide, the gender gap in life expectancy is 4.2 years, with men having an average life expectancy of 68, while women's life expectancy is 72.2 (57). However, in the more developed countries, the gender gap in life expectancy is 6.2 years, with an average life expectancy of 76.6 among men and 82.8 among women, this gender gap declines to 4 years, with an average life expectancy of 67.1 among men and 71.1 among women in less developed countries (57).

Similarly, at the age of 60, the average worldwide life expectancy for men is 18.8 years, vs. 21.6 years for women (56). However, life expectancy at this age, which on average is 20.9 years for men and 24.7 years for women in the most developed nations, decreases to 17.9 and 20.1 years (respectively) in the less developed nations, and to 16.7 and 18 years (respectively) in the least developed nations (56).

A similar phenomenon can also be seen in sex ratios—the number of men per one-hundred women. In late life, these ratios are lowest in the more developed nations and higher in the less and least developed nations. For instance, for people aged 80 and older, in the year 2017, this ratio worldwide was 64 men per 100 women. In Europe, this ratio was 53 men per 100 women, while in Asia and Africa it was 71 men per 100 women (56). The gaps in sex ratios widen with aging. For example, in the US in 2016, while the sex ratio for ages 65 and older was 79 men for every hundred women, this ratio was highest for ages 65–74 (with 88 per 100), lower for ages 75–84 (76 per 100), and lowest for ages 85 and older with only 53 men per 100 women (58). Although gender gaps in life expectancy are currently higher in developed nations and lower in less developed nations, in general, data from the last 35 years show a trend of increasing gender gaps in life expectancy in the less developed nations in favor of women and a narrowing of the gap in the developed nations (59, 60). The trend of change in the less developed nations is a result of improvements in life conditions, medicine, and increasing adoption of healthier lifestyles among women, while the decreasing gaps in life expectancy in the developed nations can be explained by the increase in percentages of women adopting unhealthy life styles, such as smoking and alcohol consumption, which are particularly harmful to women's health (61, 62). A more recently documented threat to women's health and survival in these countries is the so called “obesity epidemic” which is more prevalent among women (63). If other social conditions and risk factors for men remain stable, the spread of these risk factors among women may increase their morbidity and mortality and further reduce gender discrepancies in life expectancy in the future.

### Health and Function

Gender differences in health have been reported in several domains. Since the 1970's, biomedical literature has shown that women suffer from higher morbidity than men due to acute and chronic physical and psychiatric diseases (8, 64–68). These differences in morbidity held even when conditions associated with reproduction were excluded (64, 66).

In accordance with these reports, evaluations of functioning in old age also reveal significant discrepancies in favor of men. Disability is evaluated by measures of difficulties in performing personal care related activities of daily living (ADL) including basic personal activities, such as eating, getting dressed, washing, and using the toilet, and instrumental activities of daily living (IADL), such as home maintenance activities, managing money, walking and climbing stairs. “Life expectancy with disability” is the measure used to assess functioning throughout the life course. According to evaluations based on this measure, women on average live more years with disability than men, and the years lived with disabilities accumulate in old age. Based on data from the early 1990's, Jacobzone (3) calculated the proportion of older adults from the age of 65 who do not need significant help with at least one activity of daily living (ADL). He found that in many of the developed nations a significant discrepancy existed between the genders in favor of men. For example, the ratio of a person's life lived without disabilities in Canada was 85 for men but only 78 for women, in Japan these figures were 92 vs. 87, and in France, 94 vs. 90, respectively. Smaller gender discrepancies were presented for residents of all European countries in the year 2000 (5). For instance, in Italy from the age of 65, men had an expectancy that 92.1% of their lives would be lived free of disability, while women could expect only 90.6%. The same figures for France were 91.3% for men vs. 89.5% for women, and in Israel 91.2 vs. 88.8%, respectively. Similarly, various studies confirm that women in old age suffer more than men from limitations in physical functioning, such as ADL and IADL, and frailty (1, 4, 6, 69). In conclusion, data from various sources indicate that although women have more years of life, they also live more years with disabilities and these difficult years tend to occur during their old age. Such findings naturally raise the question: If women experience more health and functioning problems than men, how is it that they live longer?

Literature provides some explanations for this interesting paradox. One of them is found in Legato's presented data, which indicate that women are physiologically more resilient than men (70). This argument can also be derived from the way nature compensates for gender differences in survival, with a sex ratio at birth of 105 males compared to 95 females (71). Another more established explanation is based on the differences in chronic diseases and other health conditions suffered by each gender. While men suffer more than women from cancer and heart disease, which are key factors of mortality, women, in turn, have higher rates of chronic conditions, such as arthritis, depression, osteoporosis and related fractures. Such diseases cause suffering but threaten life less than cancer and heart disease do (8, 59, 67, 68, 72–76). Furthermore, even when suffering from the same diseases, such as heart disease, stroke, and arthritis, women are more likely than men to exhibit disability (63). In addition, at age 60 and over, men have greater incidence of hearing loss, injuries resulting from falls, and neck and back pain, while women suffer more than men from vision loss, depressive disorders, osteoarthritis, urinary incontinence and frailty (1, 74). The incidence and prevalence of dementia is also higher among women than among men, partially due to women's greater longevity, and the increased chance of dementia onset with aging (1, 74).

Another frequent explanation of the gender paradox in morbidity vs. longevity is based on gender differences in lifestyle, especially habits of smoking and alcohol consumption, which are strongly related to mortality, and for years have been more prevalent among men. According to a comparative study of smoking and alcohol-related deaths in 30 European countries, there is an excess of all-cause mortality among men, but the statistics vary among European countries. The ratios of men's deaths are higher in Eastern European countries compared to Western European countries. For example, alcohol-related mortality typically accounts for 20–30% of the gender gap in Eastern Europe, but decreases to 10–20% elsewhere in Europe (62).

While smoking and alcohol consumption are considered risks mainly affecting men's morbidity and mortality, the current obesity epidemic is more prevalent among women and is of special threat to women's health and functioning. For example, in a US study of 5,888 people over the age of 65, researchers found that women's odds of functional limitations were 83% higher than men's odds of the same age, with above 30% of the gender gap in disability, explained by women's obesity, and about 13% by arthritis (63). The differences in the types of chronic diseases and other health conditions suffered by men and women, as well as the differential prevalence of behavior-related risk factors can partly explain the objective reports regarding gender differences in life expectancy and life expectancy with disability in old age.

In response to the question of what happens to gender gaps in old age regarding health and disability, considering the natural biological selection that occurs in late adulthood along with social changes, the findings of some researchers in developed nations provide a partial answer. Verbrugge (66, 67) reported in a number of studies that the gender gap narrows in old age with regard to frequency of illnesses, drug consumption, and use of health services. Support for diminishing gender differences with aging in protective health behavior and in the utilization of health services in old age were also reported in Israeli studies (77, 78). Some studies that distinguished among various age groups of elderly persons indicate that gender differences are maintained in old age with a tendency to diminish only after the age of 80 (10, 79). Regarding gender differences in functional disabilities and depressive symptoms, most studies show that gaps to the disadvantage of women continue to exist in late life (63, 74, 80–83). Furthermore, while some research indicates that such gender gaps vary across studies [e.g., (82)], other studies indicate that gender gaps in some diseases, such as functional disabilities, hypertension and depression increase with aging, to the disadvantage of women, even after adjusting for age and survivorship status [e.g., (83–88)].

It appears that the question regarding gender-related gaps and the process of convergence or divergence in health and function in old age must be presented in a different way that takes into consideration what happens at the various stages of prolonged old age. It seems that in some aspects the gender differences diminish and even disappear with aging, for example life expectancy and health behaviors, but this happens only in very old age. In other aspects, such as functional disability and depression, gender differences in favor of men continue to exist.

## Gender Differences in Well-Being

On the macro level, differences in objective indicators of quality of life, such as life expectancy, life expectancy with disability, education and income across whole nations and across social groups are also expressed in subjective evaluations of general well-being, as well as in specific domains of well-being. Regarding health, generally subjective evaluations of health across countries are in accordance with the more objective data on gender differences in health and number of years lived with disabilities. For instance, the ratios of people reporting being in poor health are higher in low income nations, such as Sub-Saharan African countries than in the wealthier countries of Western Europe (8). However, in old age, gender gaps in self-rated health to the disadvantage of women exist in almost all countries and across geographical regions. In a comparison of gender gaps in self-rated health across various age groups, Boerma et al. found that 21.1% of women in comparison to 17% of men, ages 60–69, reported poor self-rated health, this gap increased in the 70–79 age group, with 30.6% for women and 22.8% for men, but decreased slightly for the 80–89 age group, with 34.2% for women and 26.9% for men (8). As can be expected, such gender gaps in self-rated health among older people also vary across nations. In some Western countries, such as the UK and US these gaps almost disappear, especially among people with the highest educational level and those who maintain an active lifestyle (89). Following their comparative study, Adjei et al. conclude that the largest contributors to gender equality in health among the elderly are equality in higher education and time spent on an active lifestyle (89).

Happiness and satisfaction with life are general measures often used for evaluation of self-perceived well-being across and within countries. In many countries, women are more likely to rank themselves lower than men on these measures (10, 12). However, similar to self-rated health and other indicators of subjective well-being, gender gaps in life satisfaction in old age vary across countries. Such differences are not explained only by gender gaps in health and functioning but also by societal effects. For instance, results of a cross-national study indicate that the sizes of gender gaps in indicators of subjective well-being vary with the extent of societal gender inequalities in personal resources, such as education and income, as well as with the cultural attitudes (norms and beliefs) regarding gender equality in various nations (90).

Another facet strongly related to subjective well-being (SWB) is mental health. Women's biological features, such as hormonal changes, along with their disadvantages in societal areas of life (socioeconomic status, gender-related social roles) extend to gender differences in mental health. Thus, women tend to report being more moody than men (91), more worried (92), more anxious (93, 94), and more stressed and depressed (11, 68, 82, 87, 95–100). Many studies have also shown that women score lower than men on psychological indicators of well-being and coping resources, such as self-esteem (10, 99, 101–104), will-to-live (9, 11), and self-efficacy (23).

An additional, central aspect of well-being is social acceptance and involvement. Being social creatures, one of the most important conditions people need for maintaining general well-being throughout the life course is having a social life in the form of sense of belonging, strong social ties and social support. However, the gender difference in life expectancy and the tendency of women to marry older men creates a worldwide situation where significantly more elderly men are married, while more elderly women are widowed and living alone (56, 105). This trend of elderly women living alone is especially noticeable in Western countries, and is becoming increasingly common in rural areas of developing countries due to work-related migration of young people to urban areas, leaving their parents behind (106, 107).

Social isolation and feelings of loneliness among older people are among the most disturbing global problems due to their negative implications for the health of aging individuals and for society as a whole. Feelings of loneliness are associated with cardiovascular diseases, rapid decrease in cognitive functioning, depression, malnutrition, and suicide among older people (108–112). In their book entitled “Loneliness: Human Nature and the Need for Social Connection” (113), Cacioppo and Patrick discuss and illustrate, such associations by presenting the role of feelings of loneliness as a central regulatory mechanism in human physiology, negatively affecting stress hormones, immune functions, cardiovascular functions, and reported risky health behaviors. It thus appears that feelings of loneliness not only negatively affect health behaviors, but are also related to detrimental physiological processes.

The scope of reported loneliness and social isolation among older persons ranges from 5 to 40% worldwide, and some researchers report that both isolation and loneliness are more prevalent among women than among men (36, 107, 114–116). Furthermore, loneliness seems to be contagious, spreading much more easily among women (117).

### Well-Being, Sexism, and Ageism

Negative stereotypical attitudes toward women followed by social discrimination—known as sexism—make a significant contribution to women's social disadvantages in education, income, and participation in the labor force, as well as to the gender gaps in health and functioning. Sexism contributes to discrimination against women and to their marginalization throughout the life span as in the case of employment, which leads to women's lower socio-economic status. The gender gaps in health and functioning are influenced by women's lower socio-economic status, and affect it in turn—a cyclical effect. Similarly, elderly people suffer from negative stereotypes, termed ageism, and from the consequences of this social classification including isolation and exclusion (118, 119). Elderly women suffer from both of these socially induced disadvantageous outcomes—sexism and ageism. All of the presented disadvantages for women in socioeconomic status, social roles, health and disability are accumulated along the life course and are significantly more harmful in old age with negative consequences for women's psychological functioning and SWB.

Personal coping resources help to moderate the negative effects of age-related losses on SWB, including the effects of losses in health and functioning (120). Older women score lower than men on psychological coping resources including resources, such as self-efficacy, mastery, and control over life (10, 121, 122). It is, therefore, not surprising that women also tend to rank themselves lower than men on cognitive and emotional measures of general well-being, such as self-rated health, satisfaction with life, happiness and will-to-live (9, 10, 15, 68, 97, 121, 123). In addition, women are less likely than men to wish to prolong their lives in difficult illness conditions by the use of life-sustaining treatment, and in general, women have a significantly weaker desire to continue living than men (9, 124). This significant gender difference in the will-to-live was found repeatedly in a number of studies of elderly people living in Israel (9, 125, 126).

From a practical point of view, subjective measures of well-being are important tools due to their diagnostic value in evaluations of well-being in general, and in specific domains of life, and due to their prognostic value in predicting morbidity and even survival in old age (15–17). In a meta-analysis of 300 empirical studies on gender differences in SWB, where SWB was evaluated by a variety of measures, Pinquart and Sorensen (127) found that not all measures of well-being demonstrate consistent gender differences. However, their final conclusion is that although in some studies gender differences in SWB were small, they continue to exist in old age to the disadvantage of women.

## Summary and Conclusions

The purpose of this paper was 2-fold: First, to present gender differences in health and well-being along with societal and lifestyle factors which contribute to these differences from a worldwide perspective. Secondly, the aim was to address the question of whether the well-established gender gaps in health and well-being diminish in old age, in view of the many biological, filial, and societal life changes faced by both genders in old age.

These issues were presented by focusing on gender differences in quality of life outcomes including life expectancy, health, functioning and subjective well-being, and their trends of change during the aging process. The paper also included descriptions of societal developments in acquisition of personal resources and lifestyle-related behaviors influencing these outcomes throughout the life course and especially in old age.

National and international comparisons indicate that women have an advantage over men in life expectancy, but they are disadvantaged in almost all dimensions of quality of life related to health, functioning and subjective well-being. Yet, there are indications that personal, family and national orientations regarding gender roles influence gender equality in health. For instance, based on a multinational European study, Palencia et al. (128) report that gender inequality in self-perceived general health is highest in traditional Southern European countries, while not significant in most dual-earner and market-oriented countries. Thus, although somewhat elevated in Western countries, societal structural and cultural barriers to women's equality in social status and personal resources continue to exist worldwide, especially in developing nations, where the process of change toward increased gender equality has only just begun or has not yet started.

Among current cohorts of older adults, women suffer more than men from disability, loneliness, and depression. Generally, women also rank lower on indicators of subjective well-being, and have a weaker will-to-live. So, do the gender gaps in health indicators diverge or converge in old age? Some international data indicate trends toward gender convergence regarding life expectancy and in some aspects of health and well-being, but only among the oldest-old and/or people with high level of education and active lifestyle. However, the gender gap in years of life with disability continues to exist into late life, to the disadvantage of women.

Regarding future cohorts of older adults, the response to the divergence vs. convergence question cannot yet be conclusive because trends having opposite influences on gender gaps in health and well-being are currently being observed. We can, however, speculate that the intensity of influence of these two different social processes on health and well-being may indeed differ over time due to differences in their pace of change, and in the way they affect health and well-being in late life. Gender equality in education, participation in the workforce and income is gradually increasing, and cracks are appearing in women's glass ceilings. The positive effects of these processes on health and well-being are relatively slow and indirect. At the same time, the increase of ratios of women involved in risky health behaviors (such as smoking, drugs and alcohol consumption, and unhealthy diets resulting in obesity) seems to be more rapid with direct effects on women's physical and mental health. This analysis leads to the inference that if other factors remain constant, in the near future, women's advantage in life expectancy in the more developed nations is likely to decrease, while the number of years that older women live with physical or mental limitations will most likely increase.

The variability in gender gaps in health and well-being across countries and in the pace of changes in these gaps over time in the various countries indicate that such gender disparities cannot merely be explained through genetic differences; instead, it appears that societal, cultural and behavioral aspects play an important role in affecting the changes in gender inequalities. Hence, in order to increase gender equality in health and well-being, societies should take action directed toward preventing and/or limiting the current negative trends by developing and implementing suitable interventions. These interventions should encourage a reduction of risky lifestyles, such as unhealthy eating habits, smoking and alcohol consumption, and should be provided to both genders and all age groups, but focus especially on females, who are more vulnerable than males to the detrimental effects of these behaviors.

Concomitantly, societies should enhance and intensify the current positive developments in national policies toward increased gender equality by removing barriers that bar women's access to socially valued personal resources and means for personal development, such as education, income, participation in the work force and in high positions. Societies should also become more involved and active in changing current cultural perceptions regarding gender-related social roles, as well as sexism and ageism. Such positive social initiatives will help to stop the vicious cycle linking negative stereotypes of women with their current social status, thus leading directly and indirectly to improvements in women's health and well-being throughout life and especially in old age. Finally, considering that women comprise more than fifty percent of the population, the empowerment of women and promotion of gender equality in health and well-being will not only benefit women, but societies at large.

## Author Contributions

SC performed the literature review and wrote the manuscript.

### Conflict of Interest

The author declares that the research was conducted in the absence of any commercial or financial relationships that could be construed as a potential conflict of interest.
